# A consolidated framework for implementation research (CFIR) guided exploration of key informant perspectives on establishing a pharmacist-led anticoagulation service in primary care: a qualitative study

**DOI:** 10.1007/s11096-024-01830-x

**Published:** 2024-11-19

**Authors:** Safaa Alshihab, Mohamed Izham Mohamed Ibrahim, Manal Al-Zaidan, Muhammad Abdul Hadi

**Affiliations:** 1 Department of Clinical Pharmacy and Practice, College of Pharmacy, QU Health Sector, Qatar University, Doha, Qatar; 2 Pharmacy and Therapeutics Supply Department, Primary Health Care Corporation, Doha, Qatar

**Keywords:** Anticoagulation clinic, CFIR, Framework analysis, Implementation research, Pharmacists, Primary health care

## Abstract

**Background:**

Globally, pharmacist-led anticoagulation services have improved patient outcomes in secondary and tertiary care settings. However, there is a paucity of literature about establishing such services within primary care settings.

**Aim:**

This study explored key informants' perceptions regarding the systemic and procedural factors influencing development and implementation of a pharmacist-led anticoagulation service in a primary care setting.

**Method:**

A descriptive qualitative study was conducted at Qatar's largest primary healthcare institution, the Primary Health Care Corporation (PHCC). Selected key informants, including healthcare center managers, pharmacy leads, physician leads and primary care physicians with cardiology privileges, were purposively recruited. Semi-structured interviews were guided by the Consolidated Framework for Implementation Research (CFIR) and analyzed using framework analysis.

**Results:**

Elven key informants were interviewed. The participants expressed confidence in the feasibility and effectiveness of implementing anticoagulation service in primary care to address patients’ needs. Key factors (mapped to CFIR domains) included ensuring pharmacist competency (Characteristics of Individuals), establishing effective internal and external communication (Inner and Outer Setting), and addressing staffing shortages (Inner Setting). Participants also emphasized on developing standardized operational protocols and training programs (Process), as well as integrating services with secondary care (Outer Setting). Despite challenges such as staffing, participants believed the service would effectively address patient needs if adequately supported.

**Conclusion:**

The implementation of pharmacist-led anticoagulation services in primary care settings was identified as both feasible and essential for improving patient outcomes. The insights from this study can inform future initiatives aimed at enhancing anticoagulation management in primary care settings.

**Supplementary Information:**

The online version contains supplementary material available at 10.1007/s11096-024-01830-x.

## Impact statements

- Implementing pharmacist-led anticoagulation services in primary care can improve patient access and reduce reliance on secondary care.
- Ensuring adequate staff and specialized pharmacist training is crucial for the effective integration of anticoagulation clinics in primary care.
- Strong communication and collaboration between primary and secondary care are essential for the successful implementation of pharmacist-led anticoagulation services.
- Employing the CFIR framework can be effective in the pre-implementation process, enabling the identification of key influencing factors and offering a structured methodology to guide the development, implementation, and sustainability of new initiatives in healthcare.

## Introduction

Pharmacists are being increasingly integrated into cognitive services using their expertise in drug information, medication safety, and patient education to optimize patient care [1–3]. Previous studies have shown that collaborative care involving pharmacists helps to optimize anticoagulation care [4–9]. Pharmacist-led anticoagulation clinics provide better anticoagulation control, lower bleeding events, and fewer warfarin-related hospitalizations than usual care [4, 10].

Several programs have been initiated to shift this model to community-based settings, enhancing patient accessibility and reducing the burden on secondary and tertiary care facilities. Evidence assessing these services in primary care settings indicates that collaborative care with pharmacists and primary care physicians result in better anticoagulation control and higher patient satisfaction than usual care [11–17].

In Qatar, evidence suggests that direct oral anticoagulants (DOACs) are not consistently prescribed appropriately [18]. In addition, the management of patients on warfarin in primary care settings is often suboptimal [19]. These findings suggest that specialized anticoagulation services within primary care settings are needed to address the existing challenges and improve anticoagulation care considering the benefits of personalized management, enhance patients’ accessibility and continuity of care.

Implementing new services in the healthcare sector can be complex because it is multifaceted and involves many stakeholders, such as patients, healthcare providers, and regulators. Implementing an anticoagulation clinic in a primary care setting is deemed more complex because of the complexity associated with anticoagulation management itself such as the need for individualized therapy, frequent monitoring, and careful consideration of drug-drug interactions particularly in elderly patients with polypharmacy [20]. Therefore, adopting a structured and comprehensive approach is important to address the complex issues in implementing such interventions.

This study is a part of a larger project aiming to evaluate the rationale and feasibility of establishing pharmacist-led anticoagulation clinics in primary care settings in Qatar with the primary aim of enhancing patient outcomes [19, 21]. Using Consolidated Framework for Implementation Research (CFIR), this study reports systemic and procedural factors influencing development and implementation of pharmacist-led anticoagulation service from the perspective of healthcare professionals and mangers.

### Aim

This study aimed to explore key informants' perceptions regarding the systemic and procedural factors influencing the development and implementation of a pharmacist-led anticoagulation service in a primary care setting.

### Ethics approval

Ethical approval was obtained in 2023 from the Institutional Review Boards (IRB) of the PHCC (BUHOOTH-D-23-00011) and Qatar University (QU-IRB 1945-EA/23).

## Method

### Study design

A descriptive qualitative study was conducted and reported according to the recommendations of the consolidated criteria for reporting qualitative research (COREQ) [22]. The processes of data collection and analysis was informed by the Consolidated Framework for Implementation Research (CFIR), as it offers a holistic lens to examine the complex interplay between factors that impact implementation outcomes. The core CFIR domains, including intervention characteristics, inner and outer settings, characteristics of individuals, and process, provide a structured framework to explore the multifaceted dynamics of introducing a new service [23, 24].

### Study setting

The study was conducted at Qatar’s largest primary healthcare institution, the Primary Health Care Corporation (PHCC). The PHCC plays an important role in delivering primary healthcare services to the population through a network of 31 health centers focusing on preventive care, health promotion, and managing common health issues [25]. The organization is solely funded by the government and provides services to both nationals and residents [26]. Each health center includes a pharmacy service managed by pharmacists who are responsible for foundational and cognitive services including medication dispensing, patient counseling, medication reconciliation, and medication review services. In addition, PHCC pharmacies provide a medication home delivery service that was introduced in 2020 [27].

### Participant sampling and recruitment

A purposive sampling strategy with maximum variation was applied to identify a broad range of key informants who may play a significant role in the development, implementation and operation processes [28]. Four main groups of key informants were identified: managers of primary health centers, pharmacy leads, physician leads, and primary care physicians with cardiology privileges. All potential key informants were invited to participate in face-to-face, semi-structured interviews through an email. Participants were recruited until data saturation was reached when predetermined codes were sufficiently reflected in the collected data [29].

### Data collection

A semi-structured interview technique was adopted. The interview questions (Online Appendix S1) were guided by the original CFIR to comprehensively examine the determinants that potentially influence the implementation of pharmacist-led anticoagulation service at the PHCC and to adapt the intervention to make it ‘fit for purpose’ within the local context before implementation [30]. Two academics (MIMI and MAH) experienced in social pharmacy and implementation research reviewed the interview guide to ensure its clarity and alignment with the research objectives. Two pilot interviews with two pharmacy leads were conducted to assess the appropriateness of the questions and address any potential challenges that might arise during the interviews. Following the pilot interviews, the sequence of questions was slightly altered, and a couple of questions were reworded slightly. The two interviews were included in the data analysis because of the richness of the responses.

The interviews were conducted by a female research pharmacist (SA) who had received training in qualitative research as part of her postgraduate studies. The research pharmacist was not employed by the PHCC and was not involved in providing care to patients at the PHCC. The interviews were conducted at the participants’ workplace from August to October 2023 in either Arabic or English depending on the participant’s preference. The interviews lasted between 20 and 52 min.

### Data analysis

Audio-recorded interviews were transcribed in a naturalized transcription style [31]. Careful proofreading was performed twice before the final transcripts were analyzed to ensure transcription accuracy. The researcher (SA) employed a deductive approach to categorize and examine the data based on predetermined codes [32]. The original CFIR domains, constructs and sub-constructs, and the CFIR codebook template were used as starting points to guide the analysis process [24, 30].

A framework analysis technique was employed to analyze the data collected from the interviews. The framework analysis was conducted in five stages [33, 34]:

1. *Familiarization*: The raw data were thoroughly reviewed to gain an overall understanding and to identify key ideas and themes, with attention to the CFIR constructs.
2. *Identifying a thematic framework*: Based on the CFIR, we identified the main recurrent themes and grouped them into broader domains. A detailed index was developed to categorize the themes and subthemes for further analysis.
3. *Indexing*: The thematic framework, based on the CFIR domains and constructs, was applied to all raw datasets. The researcher read each phrase and statement in the transcripts and labeled them with the appropriate codes derived from the index.
4. *Charting*: The data were reorganized into a thematic matrix, where each case was plotted against the main themes and subthemes, ensuring that the participant narratives were preserved within the context of the CFIR constructs.
5. *Mapping and interpretation*: Finally, the chart was used to analyze relationships between themes, identify shared concepts, establish typologies, and map the data across categories. This allowed us to develop a comprehensive interpretation of the findings within the CFIR framework.

The research team regularly met to discuss the data analysis process with experienced qualitative researcher (MAH) who checked the coding and mapping processes Codes were compared across transcripts to ensure consistency and trustworthiness of the findings [35].

## Results

### Participant demographics

Twelve participants were initially invited to participate in this study. Two did not respond, and another two could not participate because of time constraints. Three additional interviews were carried out to confirm data saturation, ensuring that the data sufficiently represented the pre-defined codes from the CFIR framework. Thus, 11 individuals were interviewed. Table 1 presents the characteristics of the participants.

**Table 1:** Characteristics of key informants

Code	Position
P1	Pharmacy lead
H1	Manager of health centre
C1	Primary care physicians with cardiology privilege
P2	Pharmacy lead
H2	Manager of health centre
P3	Pharmacy lead
C2	Primary care physicians with cardiology privilege
F1	Physician lead
H3	Manager of health centre
F2	Physician lead
P4	Pharmacy lead

### Thematic findings

The themes that emerged during the interviews were mapped according to the CFIR domains and collectively described the feasibility, barriers, and facilitators of implementing the service in primary care (Table 2).

**Table 2:** Main CFIR domains and constructs identified in interviews

Domain	Constructs	Short description*
Intervention characteristics	Evidence strength and quality	Stakeholders’ perceptions of the quality and validity of evidence supporting the belief that the intervention will have desired outcomes
	Relative advantage	Stakeholders’ perception of the advantage of implementing the intervention versus an alternative solution
	Complexity	Perceived difficulty of the intervention, reflected by duration, scope, radicalness, disruptiveness, centrality, and intricacy and number of steps required to implement
Outer settings	Cosmopolitanism	The degree to which an organization is networked with other external organizations
Inner settings	Readiness for implementation	Tangible and immediate indicators of organizational commitment to its decision to implement an intervention
	Compatibility	The degree of tangible fit between meaning and values attached to the intervention by involved individuals, how those align with individuals’ own norms, values, and perceived risks and needs, and how the intervention fits with existing workflows and systems
	Networks and Communications	The nature and quality of webs of social networks and the nature and quality of formal and informal communications within an organization
	Relative priority	Individuals’ shared perception of the importance of the implementation within the organization
Characteristics of individuals	Individual stage of change	Characterization of the phase an individual is in, as he or she progresses toward skilled, enthusiastic, and sustained use of the intervention
Process	Tailoring strategies	Choose and operationalize implementation strategies to address barriers, leverage facilitators, and fit context
	Engaging	Attracting and involving appropriate individuals in the implementation and use of the intervention through a combined strategy of social marketing, education, role modeling, training, and other similar activities

*Adapted from The Consolidated Framework for Implementation Research construct descriptions [22]

### Intervention characteristics

This domain presents key informants’ perceptions of a pharmacist-led anticoagulation service in primary care.

#### Evidence strength and quality

Most participants were unaware of research evidence supporting the effectiveness of pharmacist-led anticoagulation service in primary care. However, a few participants cited positive evidence of pharmacist-led anticoagulation clinic outcomes implemented in the secondary care setting, particularly at the HMC, the main secondary health care provider in Qatar.

“We know it is available at HMC and already implemented. Personally, I did not work on it, so I have no information about the outcomes, but I'm sure it has a positive impact.” P4

“The patients were satisfied with the convenience of walk-in services. Those I have treated at Hamad are doing well.” C1

#### Relative advantage

Many participants emphasized the necessity of specialized clinics, indicating that the current practice of warfarin management in primary care is inefficient. They attributed this inefficiency to three main factors: reluctance of some family physicians to treat patients on warfarin and would prefer to refer them to secondary care; patients primarily visiting primary care for medication refills without having a clear treatment plan; and inconsistent adherence to warfarin dispensing protocols by pharmacists.

“As family physicians, we do not have extensive background knowledge about anticoagulation management; therefore, we often have to refer the patient.” F1

“It [warfarin] is often prescribed by specialized clinics like cardiology at Hamad and family physicians in primary care only refill the prescription without making any modifications.” P4

The participants identified several advantages of the proposed model during the interviews. Many interviewees indicated that the development of a specialized anticoagulation clinic at the PHCC is anticipated to significantly improve patient accessibility to healthcare services, which would save patients both time and effort. In addition, many respondents highlighted the advantage of reducing the burden on secondary and tertiary healthcare facilities.

“This is easier for patients in terms of time and accessibility. Hamad [Secondary care provider] has a limited number of hospitals, whereas there are 31 primary care centers distributed across Qatar.” P4

Some interviewees emphasized the importance of incorporating a clinical pharmacist into primary care to address complexities of anticoagulation therapy given the high risks of drug-drug and drug-food interactions, and the need of frequent monitoring especially among elderly patients who are often on multiple medications.

“Patients on warfarin are usually on polypharmacy, so their management is complicated in which the pharmacist-led clinic would be beneficial.” P2

Several interviewees expressed concerns that the new clinic would add pressure to primary care, especially with existing staff shortages and the need to reassign staff from their regular duties. Additionally, a few participants emphasized the importance of ensuring competency of pharmacists to deliver high quality patient centered care.

“I cannot see any serious disadvantages of that [pharmacist-led anticoagulation clinic] because it is more beneficial. The only thing is to have a very competent pharmacy team.” C2

“It could be the workload, especially since we have shortages of staff, it could be one of the disadvantages.” H2

#### Complexity

The participants had diverse perspectives on the perceived difficulty of the intervention, considering factors such as the complexity of the intervention itself, integration of the service into the usual workflow, number of steps involved in the clinic execution, and complexity associated with the scope of practice of pharmacists. Many participants agreed that implementing a pharmacist-led anticoagulation model in primary care is a high-risk intervention. Some participants found it relatively easy to integrate and adapt the service within the workflow. Others attributed the complexity of the service to the scope of practice of the clinical pharmacists in primary care in Qatar, where pharmacists currently lack legal authority to prescribe medications. They cited prior instances where internal collaborative agreements were developed within HMC to permit pharmacists to prescribe medications in specialized services. Regarding the perceived complexity of the steps needed for clinic execution, the majority agreed that it would be manageable; however, they recognized that it would still require a significant amount of time.

“There is no legal prohibition preventing pharmacists from prescribing. It depends on the internal policy… It is not complicated as it is a high-risk project, you have to study everything” P1

“It takes time, which is one thing. There are several parties, so things are difficult. I am not saying this is easy; it is difficult but achievable.” C1

### Outer settings

This domain presents key informants’ perceptions of the external environment and contextual factors in which the PHCC operates, which have a substantial impact on implementing a pharmacist-led anticoagulation clinic.

#### Cosmopolitanism

The majority of participants identified HMC as the external organization with the most significant influence on the clinic's development. They emphasized that HMC is a central body whose requirements should be prioritized by the clinic. Participants also believed that HMC would support the establishment of an anticoagulation clinic in primary care settings and valued the ongoing communication and collaboration that facilitate service implementation.

“You have to work with the cardiologist in HMC to implement one clinic and run a joint clinic.” C1

### Inner settings

This domain presents key informants’ perceptions of the internal environment and contextual factors of the PHCC that impact the implementation of a pharmacist-led anticoagulation clinic.

#### Readiness for implementation

In general, all participants acknowledged the critical role of resource allocation in ensuring successful implementation of anticoagulation service. They also acknowledged the presence of skilled and knowledgeable clinical pharmacists capable of running the clinic. However, the participants strongly emphasized the necessity of comprehensive training and practical hands-on experience to ensure readiness for managing the clinic, which may require additional funding. In addition, the majority expressed concerns about the potential shortage of staff if the pharmacists were drawn from the existing workforce, as they were already grappling with staff shortages. Moreover, some participants identified the need to assign a nurse to the clinic, which poses a challenge due to the existing shortage of nurses.

“A clinical pharmacist will be taken from the available staff, which will lead to a decrease in the number of pharmacy staff.” P2

“Nurses are very limited, the staff shortage, the main problem we face.” C1

Most participants believed that other essential components, such as physical space, INR testing machines, clinical guidelines, and the necessary database, are readily available and should not be an obstacle to the development of the clinic.

“Most clinics are new, so the issue of resources is solved except in the case of some pharmacies as a manpower, but resources, empty clinics, physicians, and INR tests are available.” P3

#### Compatibility

Most participants believed implementing a pharmacist-led anticoagulation clinic at the PHCC aligned with several corporate goals, including patient safety, patient-centered care, improving health outcomes for patients with chronic diseases, and providing high-quality services. In addition, some participants believed that this intervention was compatible with the values and work processes of the PHCC.

“According to the new operational plan and strategic department, the golden goal was to minimize referrals from primary care to secondary care. It will be an excellent idea to start the clinic at this time.” F2

#### Networks and communications

There was a consensus among the participants that they had strong internal communication both between different healthcare providers and between staff and administrators, which may support the implementation. In addition, they perceived that communication with other health centers is consistently efficient.

“We have a teamwork environment, so we do not have any issues in communications with staff, supervisor, or administrators.” P2

##### Relative priority

The participants expressed diverse views on the priority of developing a dedicated anticoagulation clinic within the organization. While some considered the clinic to be of utmost priority, many held the view that there are more critical initiatives to prioritize over the clinic such as cancer screening clinics.

“Warfarin is one of the important services, looking from the risk point of view, definitely it has a priority.” C2

### Characteristics of individual

This domain describes the characteristics of individuals involved in implementing anticoagulation clinics in primary care, including administrators, and healthcare providers.

#### Individual stage of change

Many participants demonstrated a positive attitude toward the proposed model. Furthermore, they expressed the need to advance toward better utilization of the service by incorporating all anticoagulants.

“I am not against such clinics because we have always worked with specialized nurses, nurse-led clinics, and pharmacist-led clinics will be still better.” C1

Most participants believed that healthcare providers would be flexible in scheduling appointments and referring patients to the pharmacist-led clinic. They anticipated that general practitioners would readily refer their patients to the clinic, without hesitation, for anticoagulation management. “They were highly coordinated. The referrals were excellent. From previous experience with the clinical pharmacy clinic, once they knew the clinic was operated, the referral increased.” P3

### Process

This domain emerged throughout the interviews and comprised two main constructs related to the perceptions of key informants regarding effective strategies for overcoming implementation challenges, leveraging facilitators, and ultimately ensuring successful implementation.

#### Tailoring strategies

Most participants emphasized the importance of ensuring the competency of the clinical pharmacist in managing the clinic. In addition, many participants highlighted the need for pharmacists to work under the supervision of a cardiologist.

“The only thing is to have a competent pharmacy team, it is very important.” C2

Moreover, interviewees emphasized the necessity of establishing a clear operational pathway to facilitate the workflow of the service, including determining the clinic's schedule and the type of access, whether self-referral or physician referral.

"Workflow, specifically the operational part, includes factors such as working hours, number of appointments, patient load for each pharmacist, and time spent with each patient. All these factors should be taken into consideration” P1

Some participants emphasized the importance of using clinical practice guidelines for the safe use of anticoagulants in primary care settings. They suggested that the protocol should be established in collaboration with HMC and aligned with the clinical pharmacist’s scope of practice, as determined by the PHCC and Ministry of Health and includes aspects of patient selection, guidelines for regular monitoring and follow-up, and indications for referral to secondary care.

“This protocol and policy will define the tasks that can be performed, such as referring the patient back to the doctor and setting the inclusion and exclusion criteria.” P4

Effective communication was frequently highlighted as a key factor for the successful operation of the clinic. The participants emphasized the importance of clear communication with patients to educate them about the clinic's scope and the pharmacist's role. Additionally, communication with secondary care providers and various departments within the PHCC was considered essential for the efficient implementation of the clinic’s services.

"In any project or service, communication between patients and the multidisciplinary team is crucial. This enhances workplace communication and benefits both team and patient interaction.” P3

#### Engaging

This subtheme outlines the process and strategies for engaging individuals involved in the implementation and adoption of a pharmacist-led anticoagulation clinic in primary care settings to ensure its successful establishment and operation. The participants identified several strategies for engaging patients in the clinic, such as marketing campaigns and direct communication. In addition, some participants emphasized role of secondary and tertiary care physicians in creating awareness and encouraging patients to engage with the service.

“The first thing we should do is collaborate with Hamad, so the patient from Hamad or physician from Hamad should inform the patient that yes there is a clinic.” H1

## Discussion

### Statement of key findings

This study explored the perceptions of key informants regarding the development and implementation of pharmacist-led anticoagulation services in primary care settings in Qatar. The study was conducted in the backdrop of a recent retrospective study, which highlighted serious gaps, potentially compromising patient safety, in management of patients on anticoagulation therapy [19]. The participants in this study expressed strong support for implementing the service in primary care, highlighting challenges faced by patients and healthcare providers in managing anticoagulation therapy. Key informants identified pharmacist training and effective communication as key factors for successful service implementation. Additionally, they highlighted staff shortages as the primary challenge to the successful implementation of the service in primary care setting.

### Interpretation

Acknowledging the pivotal role of clinical pharmacists in clinic operations, participants emphasized the need to ensure their competency and highlighted the need for additional training to address the complexities of anticoagulation management. Studies have shown that well-structured training programs that include both theoretical and practical components are most effective for new service implementation [12, 36–38]. A study exploring the expansion of specialized pharmacist services in Qatar highlighted the critical role of continuous professional development in facilitating this expansion. Participants attributed the successful implementation of hospital-based anticoagulation clinics, in part, to specialized anticoagulation training and courses provided to pharmacists [36].

The implementation of new services requires a clear understanding of the current workflow in order to integrate the new service smoothly into the existing healthcare system. Research indicates that involving all stakeholders in the planning phase and developing an agreed protocol including the role definition of all stakeholders can ensure that the new service enhances overall workflow rather than disrupting it [12, 36, 39]. Participants in this study perceived healthcare providers to be open and accommodating in implementing a pharmacist-led anticoagulation clinic, anticipating easy patient referrals by general practitioners and overall acceptance of the clinic’s concept. However, they acknowledged that patients might take time to fully embrace this idea. Previous systematic review indicated that resistance from patients and general practitioners could create a barrier to implementing pharmacist-led anticoagulation services in community pharmacies. This resistance may stem from concerns about the perceived quality of care provided by pharmacists [40]. Jebara et al. reported comparable findings, indicating that the introduction of advanced clinical pharmacy services in Qatar, including anticoagulation services, encountered initial resistance from physicians that was promptly overcome post-implementation [36]. Educating patients about the role of the pharmacists and the goal of the service might help to manage their expectations and improve their engagement. In addition, continuous communication among healthcare providers, different health units, and organizations must be fostered to ensure that all parties are aligned with the goals and procedures of the new service. Previous studies have highlighted the importance of regular meetings, discussions, and clear communication channels to facilitate better collaboration between the involved stakeholders [24, 41, 42]. In this study, participants valued the existing communication with specialists in secondary care, as it provided the opportunity for pharmacists to access the necessary training and collaboration with cardiologist to enhance the efficient operation of the clinic.

Participants expressed concerns about pharmacists' ability to lead anticoagulation clinics in primary care in Qatar due to the lack of a formal prescribing framework in the country. However, they cited the successful implementation of an anticoagulation clinic in secondary care, which was achieved through a collaborative agreement within the institution, as a model for similar efforts in primary care. The establishment of such clinics could serve as a crucial step toward advancing pharmacist prescribing practices in Qatar particularly with previous studies that revealed strong support from wide range of stakeholders toward the implementing pharmacist prescribing in Qatar due to perceived benefits [43–45]. These studies also emphasized the need for a systematic framework to ensure pharmacist competency through comprehensive training programs and accreditation systems.

### Strengths and weaknesses

This study has several strengths. First, engaging diverse key informants in the planning process helps to understand and address their concerns, ensuring successful implementation of the clinic. Second, using the CFIR framework to develop interview guide and the data analysis coding process ensured a systematic and comprehensive examination of all aspects of the clinic’s implementation. While the majority of studies utilizing the CFIR framework focus on the post-implementation phase to understand determinants of implementation success [46], this study is distinct in its application of CFIR during the pre-implementation phase. This early-stage use allows for the identification of potential challenges and facilitators before implementation, enabling proactive strategies to enhance the likelihood of successful outcomes. Since the original CFIR framework [24] published in 2009 was used in this study, future studies should consider using the updated framework to ensure alignment with the latest advancements in implementation research [47].

Although this study provides valuable insights into pharmacist-led clinics, it is essential to acknowledge its limitations. Excluding the perspectives of patients and external stakeholders (i.e. secondary care providers and representatives from the Ministry of Health) limits the comprehensiveness of insights. While patients' perspectives are indeed important, as they are key stakeholders in the healthcare process, the specific aim of this study was to understand internal processes, challenges and system-level factors that influence successful implementation among healthcare professionals, which may not be fully understood by patients. Future studies should incorporate patient perspectives to inform meaningful improvements in patient-centered care and ensure that healthcare services align with their expectations and outcomes. Furthermore, investigating the long-term outcomes and sustainability of pharmacist-led anticoagulation clinics in primary care settings, including their impact on patient outcomes, healthcare resource utilization, and cost-effectiveness, should be investigated before wider implementation.. This study attempted to strength the trustworthiness of the research findings by outlining detailed data collection and data analysis processes and by practicing reflexivity to consider potential biases. The interviews were conducted by a researcher not affiliated with the corporation thereby mitigating potential conflicts of interest that could have skewed the findings. However, the researcher's profession as a pharmacist might have a potential influence on the participants’ way articulated their perspectives.

## Conclusion

The findings of this study offer critical guidance for the future development and implementation of anticoagulation services in primary care, contributing to better healthcare outcomes and more efficient resource utilization. Despite some concerns, particularly around resources and legal authority for prescribing, participants believed that such a service could significantly enhance patient care by improving access and reducing the burden on secondary care. The barriers identified highlight the need for proactive strategies to address these challenges. The strategies suggested by key informants, including effective communication and specialized staff training offer actionable insights for overcoming these barriers.

## Supplementary Information

Below is the link to the electronic supplementary material.

Supplementary file1 (DOCX 23 KB)

## References

[1] Roberts AS, Benrimoj SI, Chen TF, et al. Implementing cognitive services in community pharmacy: a review of models and frameworks for change. Int J Pharm Pract. 2006;14(2):105–13.

[2] Campeau A, Duval C, Laberge M, et al. Clinical services in community pharmacies: a scoping review of policy and social implications. Int J Pharm Pract. 2021;29(2):116–25. 33729524 10.1093/ijpp/riaa007

[3] Bennett M, Goode J-VR. Recognition of community-based pharmacist practitioners: essential health care providers. J Am Pharm Assoc. 2016;56(5):580–3. 10.1016/j.japh.2016.04.566 27594108

[4] Saokaew S, Permsuwan U, Chaiyakunapruk N, et al. Effectiveness of pharmacist-participated warfarin therapy management: a systematic review and meta-analysis. J Thromb Haemost. 2010;8(11):2418–27. 20831620 10.1111/j.1538-7836.2010.04051.x

[5] Manzoor BS, Cheng W-H, Lee JC, et al. Quality of pharmacist-managed anticoagulation therapy in long-term ambulatory settings: a systematic review. Ann Pharmacother. 2017;51(12):1122–37. 28735551 10.1177/1060028017721241

[6] Entezari-Maleki T, Dousti S, Hamishehkar H, et al. A systematic review on comparing 2 common models for management of warfarin therapy; pharmacist-led service versus usual medical care. J Clin Pharmacol. 2016;56(1):24–38. 26100092 10.1002/jcph.576

[7] Kurochka I, Jadallah J, Nadpara P, et al. Community-based pharmacist anticoagulation clinic outcomes compared with physician management. Innov Pharm. 2024;15(1):8. 10.24926/iip.v15i1.5929 PMC11107966 38779104

[8] Morrison L, Nagge J. The quality of pharmacist-led community warfarin management across 2 provinces in Canada: a cross-sectional observational study. Can Pharm J. 2024;157(2):77–83. 38463172 10.1177/17151635241228228 PMC10924574

[9] Beyene K, Chan AHY, Bandreddi NST, et al. Patient satisfaction with community pharmacist-led anticoagulation management services and its relationship with patient characteristics in New Zealand. Int J Clin Pharm. 2021;43(1):154–64. 32808187 10.1007/s11096-020-01124-y

[10] Hou K, Yang H, Ye Z, et al. Effectiveness of pharmacist-led anticoagulation management on clinical outcomes: a systematic review and meta-analysis. J Pharm Pharm Sci. 2018;20(1):378–96. 10.18433/J3SQ0B 29145935

[11] Harrison J, Shaw JP, Harrison JE. Anticoagulation management by community pharmacists in New Zealand: an evaluation of a collaborative model in primary care. Int J Pharm Pract. 2015;23(3):173–81. 25250655 10.1111/ijpp.12148

[12] Shaw J, Harrison J, Harrison J. A community pharmacist-led anticoagulation management service: attitudes towards a new collaborative model of care in New Zealand. Int J Pharm Pract. 2014;22(6):397–406. 24612135 10.1111/ijpp.12097

[13] Ingram SJ, Kirkdale CL, Williams S, et al. Moving anticoagulation initiation and monitoring services into the community: evaluation of the Brighton and hove community pharmacy service. BMC Health Serv Res. 2018;18(1):91. 29415718 10.1186/s12913-018-2901-8 PMC5803888

[14] Young S, Bishop L, Twells L, et al. Comparison of pharmacist managed anticoagulation with usual medical care in a family medicine clinic. BMC Fam Pract. 2011;12:88. 21849052 10.1186/1471-2296-12-88 PMC3176160

[15] Woodill L, Bodnar A. A novel model of care in anticoagulation management. Healthc Manag Forum. 2020;33(6):247–52. 10.1177/0840470420969444 33086887

[16] Falamić S, Lucijanić M, Hadžiabdić MO, et al. Pharmacist’s interventions improve time in therapeutic range of elderly rural patients on warfarin therapy: a randomized trial. Int J Clin Pharm. 2018;40(5):1078–85. 30051222 10.1007/s11096-018-0691-z

[17] Falamić S, Lucijanić M, Ortner-Hadžiabdić M, et al. Pharmacists’ influence on adverse reactions to warfarin: a randomised controlled trial in elderly rural patients. Int J Clin Pharm. 2019;41(5):1166–73. 31493209 10.1007/s11096-019-00894-4

[18] Elewa H, El-Makaty H, Ali Z. Appropriateness of dabigatran and rivaroxaban prescribing in Qatar: A 5-year experience. J Cardiovasc Pharmacol Ther. 2018;23(2):155–61. 28978236 10.1177/1074248417731536

[19] Alshihab S, Mohamed Ibrahim MI, Hadi MA, et al. Evaluation of warfarin management in primary health care centers in Qatar: a retrospective cross-sectional analysis of the national dataset. Curr Probl Cardiol. 2024;49(4): 102427. 38301919 10.1016/j.cpcardiol.2024.102427

[20] Wadsworth D, Sullivan E, Jacky T, et al. A review of indications and comorbidities in which warfarin may be the preferred oral anticoagulant. J Clin Pharm and Ther. 2021;46(3):560–70. 33393699 10.1111/jcpt.13343

[21] Alshihab S, Mohamed Ibrahim MI, Abdul Hadi M, et al. Exploring stakeholder perceptions of implementing a pharmacist-led anticoagulation clinic in primary care settings: a cross-sectional study. J Pharm Policy Pract. 2024;17(1):2395529. 39253621 10.1080/20523211.2024.2395529 PMC11382688

[22] Tong A, Sainsbury P, Craig J. Consolidated criteria for reporting qualitative research (COREQ): a 32-item checklist for interviews and focus groups. Int J Qual Health Care. 2007;19(6):349–57. 17872937 10.1093/intqhc/mzm042

[23] Breimaier HE, Heckemann B, Halfens RJ, et al. The consolidated framework for implementation research (CFIR): a useful theoretical framework for guiding and evaluating a guideline implementation process in a hospital-based nursing practice. BMC Nurs. 2015;14:43. 26269693 10.1186/s12912-015-0088-4 PMC4533946

[24] Damschroder LJ, Aron DC, Keith RE, et al. Fostering implementation of health services research findings into practice: a consolidated framework for advancing implementation science. Implement Sci. 2009;4:50. 19664226 10.1186/1748-5908-4-50 PMC2736161

[25] Primary Health Care Corporation. Available from: https://www.phcc.gov.qa/. Accessed 26 Feb 2024.

[26] World Health Organization. Qatar Health Profile 2016. Available from: https://applications.emro.who.int/dsaf/EMROPUB_2016_EN_18927.pdf?ua=1. Accessed 26 Aug 2024.

[27] Primary Health Care Corporation. Clinics and services. Available from: https://www.phcc.gov.qa/clinics-and-services/browse-by-category/pharmacy. Accessed 26 Aug 2024.

[28] Palinkas LA, Horwitz SM, Green CA, et al. Purposeful sampling for qualitative data collection and analysis in mixed method implementation research. Adm Policy Ment Health. 2015;42(5):533–44. 24193818 10.1007/s10488-013-0528-y PMC4012002

[29] Saunders B, Sim J, Kingstone T, et al. Saturation in qualitative research: exploring its conceptualization and operationalization. Qual Quant. 2018;52(4):1893–907. 29937585 10.1007/s11135-017-0574-8 PMC5993836

[30] Consolidated framework for implementation research guide. Available from: https://cfirguide.org/. Accessed 01 Sep 2023.

[31] Bucholtz M. The politics of transcription. J pragmat. 2000;32(10):1439–65.

[32] Miles MB, Huberman AM. Qualitative data analysis: a sourcebook of new methods. Sage; 1984.

[33] Ritchie J, Spencer L, Bryman A, et al. Analysing qualitative data. Routledge, London; 1994. ISBN 9780203413081

[34] Ritchie J, Lewis J, Nicholls CM, et al. Qualitative research practice: A guide for social science students and researchers: Sage; 2013. ISBN 1446296202.

[35] Hadi MA, José CS. Ensuring rigour and trustworthiness of qualitative research in clinical pharmacy. Int J Clin Pharm. 2016;38(3):641–6. 26666909 10.1007/s11096-015-0237-6

[36] Jebara T, Cunningham S, MacLure K, et al. Health-related stakeholders’ perceptions of clinical pharmacy services in Qatar. Int J Clin Pharm. 2021;43(1):107–17. 32960428 10.1007/s11096-020-01114-0 PMC7878249

[37] Tadesse TA, Abiye AA, Endale S, et al. Challenges of anticoagulation management service and need of establishing pharmacist-led anticoagulation clinic in tertiary care teaching hospital, Ethiopia: a qualitative study. J Multidiscip Healthc. 2022;15:743–54. 35418756 10.2147/JMDH.S359558 PMC8995148

[38] Shimabukuro TT, Kramer J, McGuire M. Development and implementation of a nurse-managed anticoagulation program. J Healthcare Qual. 2004;26(1):4–13. 10.1111/j.1945-1474.2004.tb00466.x 14763315

[39] Tonna A, McCaig D, Diack L, et al. Development of consensus guidance to facilitate service redesign around pharmacist prescribing in UK hospital practice. Int J Clin Pharm. 2014;36(5):1069–76. 25108412 10.1007/s11096-014-9996-8

[40] Egunsola O, Li JW, Mastikhina L, et al. A qualitative systematic review of facilitators of and barriers to community pharmacists-led anticoagulation management service. Ann Pharmacother. 2022;56(6):704–15. 34510918 10.1177/10600280211045075 PMC9008548

[41] Pronovost PJ, Berenholtz SM, Goeschel CA, et al. Creating high reliability in health care organizations. Health Serv Res. 2006;41(4 Pt 2):1599–617. 16898981 10.1111/j.1475-6773.2006.00567.x PMC1955341

[42] Edmondson AC, Bohmer RM, Pisano GP. Disrupted routines: team learning and new technology implementation in hospitals. Adm Sci Q. 2001;46(4):685–716.

[43] Diab MI, Ibrahim A, Abdallah O, et al. Perspectives of future pharmacists on the potential for development and implementation of pharmacist prescribing in Qatar. Int J Clin Pharm. 2020;42(1):110–23. 31898166 10.1007/s11096-019-00946-9 PMC7162834

[44] Jebara T, Cunningham S, MacLure K, et al. A modified-Delphi study of a framework to support the potential implementation of pharmacist prescribing. Res Soc Adm Pharm. 2020;16(6):812–8. 10.1016/j.sapharm.2019.09.005 31522998

[45] Jebara T, Cunningham S, MacLure K, et al. Key stakeholders’ views on the potential implementation of pharmacist prescribing: a qualitative investigation. Res Soc Adm Pharm. 2020;16(3):405–14. 10.1016/j.sapharm.2019.06.009 31253499

[46] Kirk MA, Kelley C, Yankey N, et al. A systematic review of the use of the consolidated framework for implementation research. Implement Sci. 2016;11:72. 27189233 10.1186/s13012-016-0437-z PMC4869309

[47] Damschroder LJ, Reardon CM, Widerquist MAO, et al. The updated consolidated framework for implementation research based on user feedback. Implement Sci. 2022;17(1):75. 36309746 10.1186/s13012-022-01245-0 PMC9617234

