# Supplementary material for: A consolidated framework for implementation research (CFIR) guided exploration of key informant perspectives on establishing a pharmacist-led anticoagulation service in primary care: a qualitative study
Source: Int J Clin Pharm. 2024 Nov 19;47(2):335–44. doi: 10.1007/s11096-024-01830-x (PMC11919966; doi:10.1007/s11096-024-01830-x)
Supplement: Supplementary file 1 — Supplementary file1 (DOCX 23 KB) [file 11096_2024_1830_MOESM1_ESM.docx]

**Supplementary Material**

**Appendix S1. Final interview guide^1^**

**-----------------------**

**How do you perceive the current quality of OAC and warfarin management at PHCC?**

**How warfarin can be improved at the PHCC?**

**1.There is a lot of research evidence to support the role of pharmacists in anticoagulation. Have you**

**had a chance to read it?**

How do you perceive the quality and validity of evidence that support establishing a specialized

anticoagulation clinic will have a desired outcome?

**2. Do you believe anticoagulation clinic in primary settings is an effective service? Why or why not?**

Do you believe this service will work better than other alternatives?

What observed benefits you may observe? What are the advantages of this service over

another?

To what extent do current programs fail to meet patient needs? Will the clinic meet these needs?

What disadvantages does the intervention have compared to these other programs?

Is there another intervention that people would rather implement?

**3.Regardig pharmacist-led anticoagulation clinic, can this service be adapted for local contexts? Qatar,**

**PHCC, Community,,,**

How do you think the clinic should be organized?

Are there components that should not be altered?

Do you think you will be able to make these changes? Why or why not?

What can be potential challenges/barriers to its adaptation?

**4. Can this service be tested on a smaller scale within the PHCC, and can it be reversed if needed?**

**5. Do you think it would be possible to pilot the intervention before making it available to**

**everyone?**

Why or why not? Would this be helpful?

**6. How difficult do you perceive implementation of this service to be?**

How complicated is the intervention?

**7. How essential is this intervention to meet the needs of the individuals served by your**

**organization or other organizational goals and objectives?**

**6. Are patients’ needs known and prioritized by the PHCC mission**

The extent to which PHCC is successful in achieving this degree of “patient-centeredness”

**1. To what extent is staff aware of the needs and preferences of the individuals (Patients) being**

**served by your organization?**

**2. To what extent were the needs and preferences of the individuals served by your organization**

**considered when deciding to implement the intervention?**

**3. How well do you think the intervention will meet the needs of the individuals served by your**

**organization?**

o In what ways will the intervention meet their needs? E.g. improved access to services?

Reduced wait times? Help with self-management? Reduced travel time and expense?

**4. How do you think the individuals served by your organization will respond to the intervention?**

**5. What barriers will the individuals served by your organization face to participating in the**

**intervention?**

**6. Is the PHCC networked with other external organizations such as HMC?**

What do you perceive about the acceptance/support of the external organization to this service?

**7. Are there any policy, recommendations, or mandates to support the implementation of this type of**

**service?**

**8. How will the infrastructure facilitate/hinder implementation of the intervention?**

**9. What kinds of infrastructure changes will be needed to accommodate the intervention?**

Changes in scope of practice? Changes in formal policies? Changes in information systems or electronic records systems? Other? What kind of approvals will be needed? Who will need to be involved? Can you describe the process that will be needed to make these changes?

**10. How important is effective communication affect implementation of this service?**

Is there good communication between individuals, units, services, and organization levels?

**11. What is your motivation for wanting to help ensure the implementation is successful?**

**12. What kind of support or actions can you expect from leaders in your organization to help make**

**implementation successful? What types of barriers might they create?**

**13. What do you perceive about readiness towards pharmacist-led anticoagulation clinic?**

Flexibility? Acceptance from patients, physicians? Is there is qualified pharmacists to implement and manage anticoagulation clinic

**14. Do stakeholders perceive the current situation as needing change? Professionals, leaders?**

**15. How important is this intervention when compared to competing priorities of individual stakeholders, upper management, and the organization as a whole?**

What kinds of high-priority initiatives or activities are already happening in your setting? Describe activities or initiatives that (appear to) have highest priority for you (for the organization)? What kind of pressure are you feeling to accomplish this? Where is it coming from? Why?

**16. What are your perceptions about individuals’ personal traits that may affect implementation of**

**the services?**

Do you think pharmacists are competent and skilled enough to manage patients’ anticoagulation? why?

Would GP’s believe that pharmacists are competent and skilled to provide the service?

What kind of issues do you think GPs’ will have with this service?

Would GP’s want to refer patients to the clinic? Why or why not?

**17. What is your perception of an effective implementation process?**

Who is involved in the implementation?

Does the implementation effort need a champion?

What is their role in the organization and in the implementation?

Individuals external to the implementation process facilitate implementation decisions.

What kind of information is planned to collect as the intervention is implemented?

Which measures will you track? How will you track them?

**^1^** The interview guide questions were adapted from the CFIR Interview Guide Tool, available at https://cfirguide.org/guide/app/#/
